# Mesothelioma with Nonbacterial Thrombotic Endocarditis: A Case Report

**DOI:** 10.31729/jnma.6841

**Published:** 2021-11-30

**Authors:** Avatar Verma, Narendra Bhatta, Deebya Raj Mishra, Achyut Bhakta Acharya, Rejina Shahi, Sion Hangma Limbu, Srijan Katuwal, Urmila Lama

**Affiliations:** 1 Department of Pulmonary, Critical Care and Sleep Medicine, B. P. Koirala Institute of Health Sciences, Dharan, Nepal; 2 Department of Paediatrics and Adolescent Medicine, B. P. Koirala Institute of Health Sciences, Dharan, Nepal

**Keywords:** *malignancy*, *mesothelioma*, *nonbacterial thrombotic endocarditis*

## Abstract

Non-bacterial thrombotic endocarditis is a rare condition characterized by noninfectious vegetation on cardiac valves which are often associated with malignancy. It often presents with features of embolism rather than cardiac failure. These are usually seen in autoimmune conditions, disseminated intravascular coagulation, malignancy of gut and lung but has also been reported in other malignancies as well. This entity is rare but one must have a clinical suspicion of the disease especially in a patient suffering from malignancy presenting with the embolic phenomenon. In this report, we are presenting a case of non-bacterial thrombotic endocarditis in an inpatient with pleural mesothelioma, a rare malignant neoplasm arising from pleura in a 35 years old mason, and a rare association as well.

## INTRODUCTION

Non-bacterial thrombotic endocarditis (NBTE) is characterized by the presence of vegetations on cardiac valves, consisting of fibrin and platelet aggregates, and is devoid of inflammation or bacteria. NBTE is a rare condition often associated with hypercoagulable states or advanced malignancy such as adenocarcinomas. It is also associated with systemic lupus erythematosus and antiphospholipid syndrome also called Libman Sick's endocarditis.^1^ Among malignancy it is more commonly associated with mucin-producing adenocarcinoma of the gut and lung.^2^ Hereby, we report an unusual combination of NBTE and malignant pleural 'mesothelioma'.

## CASE REPORT

A 35 years old mason with cement exposure for the last 20 years and a smoking history of 20 pack years presented to the emergency department with dry cough and right-sided lateral chest pain for 2 weeks.

He was tachypneic with a respiratory rate of 26 breaths per minute. His blood pressure was in the normal range (100/60mmHg) and had a pulse rate of 110 beats per minute and oxygen saturation of 90% in room air. His trachea was central, with reduced air entry and dull percussion notes on the right chest.

His chest x-ray showed right-sided pleural effusion which on pleural fluid tapping was found to be hemorrhagic and on the analysis found to be exudative. Given the degree of respiratory distress due to massive pleural effusion, a 24 Fr chest tube was inserted and around 3L of hemorrhagic fluid was drained. Following that, there was continuous drainage of hemorrhagic pleural fluid greater than 500 ml per day. Contrast-enhanced Computed Tomography (CECT) Chest was performed and showed moderate right-sided pleural effusion with pleural thickening and nodular opacities (Figure 1a, b).

**Figure 1a:**
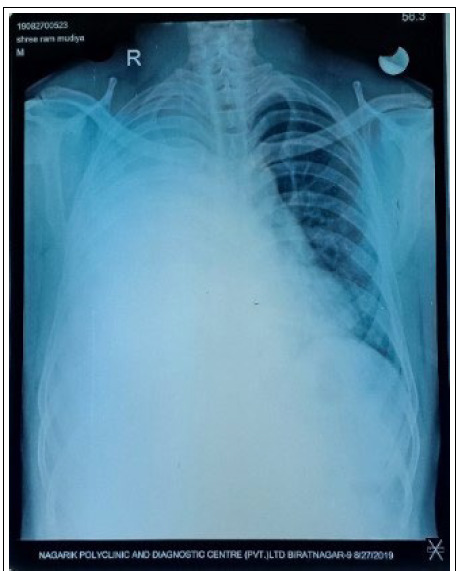
Chest x-ray; Right-sided pleural effusion.

**Figure 1b:**
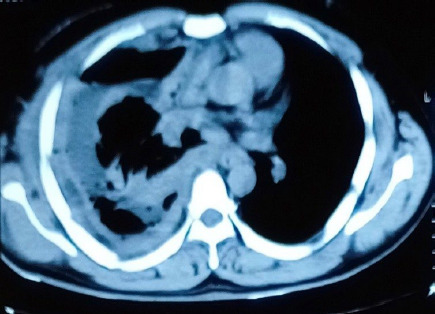
CECT Chest: Right-sided pleural thickening and nodular opacities.

Following this, the patient underwent thoracoscopy revealing multiple pleural-based nodules from which biopsy samples were taken and sent for histopathology.

On the 5^th^ day of admission patient developed sudden onset left-sided complete hemiparesis with associated difficulty in swallowing. Neurological findings were suggestive of stroke involving right anterior circulation.

**Figure 2a:**
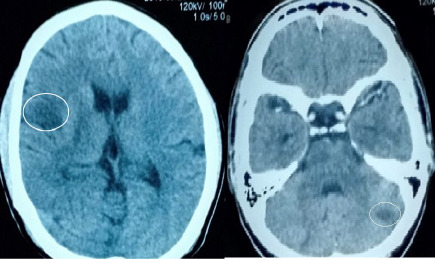
Hypodensity involving the right parietal lobe of the brain. **Figure 2b.** Hypodensity in the left lobe of the cerebellum.

An urgent CECT Head was performed that revealed non-enhancing hypodense lesions in the right parietal lobe and left cerebellum suggestive of the acute infarct. With this diagnosis of acute ischemic stroke was made (Figure 2a, b).

**Figure 3:**
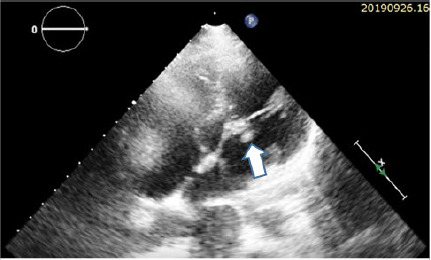
Echocardiography (subxiphoid view) showing vegetation in the anterior mitral leaflet.

Electrocardiography and carotid doppler study were normal while transthoracic echocardiography revealed vegetation of size 8.4 × 6 mm in anterior leaflet of mitral valves along with mitral regurgitation suggesting endocarditis (Figure 3).

Three sets of serial blood cultures were sent within 24 hours; 12 hours apart came negative. Workup for autoimmune disease, connective tissue disorder, coagulopathy, and HIV serology all was negative. In view of all these findings patients were diagnosed with a case of marantic endocarditis with systemic embolization for which full dose enoxaparin was started.

The patient on the 10th day of admission and 5th day of onset of stroke started improving the power of right upper and lower limbs and facial asymmetry also became less prominent. However pleural fluid drainage was continuous amounting to more than 500 ml per day. In the meantime, the biopsy report came out to be mesothelioma of epitheloid variety and immunohistochemistry was positive for calretinin and cytokeratin 5/6. On this background, a diagnosis of Malignant Pleural Mesothelioma was made. Carboplatin and Pemetrexed were given on the 12th day of admission following all standard protocols.

On the 15th day of admission, the patient developed severe abdominal pain. On evaluation Blood Pressure was unrecordable, pulse was thready. However, the abdomen was soft and non-tender. The patient and patient party were counseled regarding the possibility of mesenteric ischemia however they refused further workup and denied shifting to ICU and the patient later collapsed.

## DISCUSSION

Non-bacterial thrombotic endocarditis is a rare condition characterized by the deposition of sterile thrombi on mainly left-sided heart valves and recurrent thromboembolic events. Patients with advanced malignancy and SLE are the most common cause. Adenocarcinoma is the most frequent histologic type while lung, pancreas, gastric cancer, and adenocarcinoma of the unknown primary site are the most common causes associated with NBTE.^3^ Incidence ranges from 0.9 to 1.6%.^4^

Mesothelioma cells secrete procoagulant factors and interleukin 6, which can hasten platelet function and thrombosis while promoting inhibition of fibrinolysis, this probably explains hypercoagulable state and consecutive NBTE.^5,6^

There are few case reports where NBTE has been seen to be associated with lung neoplasm especially adenocarcinoma either preceding or following diagnosis of lung neoplasm.^7,8^ There are case reports associated with NBTE and small cell lung cancer.^9^ However we could not find any case reports associating mesothelioma and non-bacterial thrombotic endocarditis. To our knowledge, this is the first case report depicting the association of mesothelioma and NBTE.

Systemic emboli occur in nearly 50% of patients with NBTE affecting mainly cerebral, coronary, renal, and mesenteric circulations; and sudden neurological deficit being the most common presentation.^10^ Other case reports also support this finding which is in concordance with our case report^11,12^

The limitation in this case report was patient party denial in performing further workup for mesenteric ischemia which would have given us a further clinical picture of the patient.

A high index of clinical suspicion is key to diagnosis for NBTE. Demonstration of valvular vegetations on echocardiography in the absence of systemic infection in patients who are at high risk of NBTE provides strong evidence to support the diagnosis.^13^

Treatment of NBTE is difficult however treatment of the underlying cause is most important along with full anticoagulation using low molecular weight heparin. Surgical interventions are utilized when the patient either presents/ develops acute congestive failure despite the above treatment.^14^

Thus, our case report emphasizes the need to consider the possibility of NBTE in any cancer patient presenting with the embolic phenomenon.
